# Supplementary material for: Influenza Vaccination Uptake and Prognostic Factors among Health Professionals in Italy: Results from the Nationwide Surveillance PASSI 2015–2018
Source: Vaccines (Basel). 2023 Jul 10;11(7):1223. doi: 10.3390/vaccines11071223 (PMC10386716; doi:10.3390/vaccines11071223)
Supplement: Supplementary file 1 [file vaccines-11-01223-s001.zip › vaccines-2425374-supplementary.pdf]

Table S1. Study on seasonal influenza vaccination uptake among health professionals in Italy, 1990-2022.

Reference	Study Period	Setting	Health professional (HP) population under study	Type of study, sampling (s.) strategy	Measures of vaccine acceptance (VA), attitudes towards seasonal influenza vaccination (SIV)	N (Total sample size), Response rate	Findings
[1]	Nov 2009 – Jan 2010	Eight non-teaching hospitals (Naples and Avellino, Campania)	Physicians, nurses, technicians, ancillary staff	Anonymous survey, random s.	SIV uptake or willingness. Perception of SIV risks and benefits.	720, 83.3%	16.7% vaccinated; 43.6% of unvaccinated not planning to receive SIV. Mean rated SIV utility 4.3/10. Mean rated SIV dangerousness 5.7/10. 31% fear of adverse effects
[2]	2006 – 2014	Teaching hospital (Genoa, Liguria)	Physicians, nurses, other clinical personnel	Intervention study (vaccine promotion strategies), no s.	SIV uptake	3444, NA	SIV coverage fluctuating over the years, from 20% at study start to 16% at study end, peaking at 34% in 2009-10
[3]	Three flu seasons (2005-6 to 2007-8)	Teaching hospital (Palermo, Sicily)	All personnel (incl. admin. staff w/ patient contact)	Analysis of administrative data, no s.	SIV uptake	~2600, NA	Mean coverage 11.2%, declining from 14.7% at the start to 8.2% at the end. Higher rate among biologists and physicians vs nurses and other staff, and among males vs females
[4]	Sep 2010 – Oct 2010	Teaching hospital (Palermo, Sicily)	Medical residents	Anonymous web-based survey, no s.	SIV uptake in previous season. Intention to vaccinate for current season. Perception of SIV benefits for own and others' health	302, 66.9%	21.8% vaccinated previous year. 22.3% planning to vaccinate. Higher OR of intention associated w/ perception of SIV safety, efficacy, and risk for themselves, patients and general population
[5]	Nov 2009 – Feb 2010	Teaching hospital (Palermo, Sicily)	All personnel	Analysis of administrative data, no s.	SIV uptake	2267, NA	18.7% vaccinated. Higher OR associated w/ male sex, physicians and biologists, SIV in previous season
[6]	Nov 2018 – Mar 2019	Teaching hospital (Sassari, Sardinia)	All personnel	Anonymous web-based survey, no s.	SIV uptake or intention.	2270, 20.1%	30.6% coverage, 44.2% willing. Higher OR for physicians, awareness of risk for themselves and some categories; lower OR for working in surgical wards, more contact time w/ patients, difficulties in access

[7]	Oct 1990	Eleven local health care centres (5 Italian regions)	Physicians, nurses, pharmacists	Anonymous survey; s. strategy not reported	SIV uptake in previous season and intention for current season. Perception of SIV efficacy and risks	1129, 99.5%	18.8% vaccinated previous year (35% pharmacists). 27.3% planning to vaccinate. >90% physicians and pharmacists convinced of SIV efficacy; 24.2% nurses contrary or dubious. Intention mostly related to perception of own risk from disease
[8]	Starting from the 2015-2016 campaign	Gemelli Policlinic Hospital, Rome	HP	quasi-experimental study	evaluate the efficacy of different strategies implemented during the last four years (2015-2019)	almost 4000 HCWs each year	Increasing SIV from 6% in 2015-2016 to almost 22% at the end of 2018-2019. The overall number of vaccinated HP increased, especially at younger ages. OSV[3] strategy led to better results; physicians had higher SIV than nurses and others.
[9]	2012 – 2013	Multipurpose study from National Institute of Statistics	All personnel	Face-to-face interviews, census samples	SIV uptake in the past 12 months	5823, NA	18.1% vaccinated. Higher OR associated w/ male sex, older age, chronic diseases, and poor self-reported health
[10]	2018-2019 and 2017-2018 flu seasons	44 out of 50 the Bari Policlinico hospital operative units	HP	Cross-sectional study	For the 2018/2019 influenza season, OSV[3] was offered directly in 44 units of the Bari Policlinico hospital (50 units, 3,397 HP). The hospital granted the HP access to the vaccination clinic during October and December 2018.	[SIV in 798 HP - 2018/19 season vs 482 HP - 2017/18 season]	2018/19 SIV 20.4% (n = 798) > 2017/18 SIV 14.2% (+6.2%). The highest VA among physicians (33.4%), followed by other HP (23.8%), auxiliary staff (8.6%), and nurses (7.2%). 284 HP (36.5%) vaccinated at on-site sessions. Vaccine uptake associated with male gender and working where active vaccination offer in place. OSV[3] improved VA by 44% compared to the previous season.
[11]	Oct 2010 – Nov 2010	Empoli and Pistoia LHUs[1] and Careggi teaching hospital (Tuscany)	All personnel	Anonymous survey, convenience samples	SIV uptake or willingness. Opinions about SIV	1996, NA	SIV rate 45.1% among physicians, 15% nurses, 17.7% other HP. Higher OR associated w/ male sex, older age, chronic resp. diseases and diabetes, living w/ a person w/ comorbidities, previous SIV, poor self-reported health. Avoidance

						related to low perception of risk infection.
[12]	Oct 2010 – Apr 2011	Empoli and Pistoia LHUs[1] and Careggi teaching hospital (Tuscany)	All personnel, including nonclinical	Anonymous survey, convenience samples	SIV uptake last 3 years. Opinions about SIV	11369, 1975 respondents	SIV coverage ~18% during 3 years. Higher VA among males, physicians, chronic diseases, poor self-reported health, at-risk contacts. Self and family protection main drivers of VA; perception of low severity and risks of vaccine main reasons for refusal
[13]	Dec 2019	Seven hospitals (Friuli-Venezia Giulia)	All personnel	Survey of hospital managements, no s.	SIV uptake	12627, NA	Mean SIV coverage 24.9% (range 17% – 33.3%). Best results in hospitals distributing kits in units for on-site administration.
[14]	Mar 2013	Thirty hospitals (Sardinia)	All personnel	Survey of hospital managements, no s.	SIV uptake	12977; SIV data from 23 hospitals	20 out of 23 hospitals reported SIV coverage <16%, 9 of which reported less than 6%.
[15]	Oct 2007	Rivoli hospital (Piedmont)	All personnel	Anonymous survey, no s.	SIV uptake previous 2 years. Reasons for adherence or refusal.	773, 43.6%	25.8% coverage, higher for physicians (> 50%) than other HP (~20%). Self-protection main reason of VA; refusal mostly motivated by low belief in SIV efficacy.
[16]	2016-2019	A northern Italian university	Third-year healthcare students	Cross-sectional study, anonymous online self-administered questionnaire	SIV uptake in three campaigns. Reasons for adherence or refusal.	352 / 392, 90%	Self-protection as main reason for adherence (87.5%), perception of influenza as non-threatening (24.4%) as main reason for refusal. Statistically significant associations with adherence to 2018-2019 campaign: being a nursing/midwifery student and agreeing or being undecided about mandating vaccination in health facilities. Low vaccine uptake, but good knowledge of the flu-driven risks.
[17]	Jan 2011	GP[2] training course of West Sicily	GP[2] trainees	Anonymous survey, no s.	SIV uptake last five years. Reasons for adherence.	105, 76.2%	Coverage 26.2% previous year, 18.7% current year. Higher OR for perception of high infection risk.

[18]	Sep 2010 – Oct 2010	Four post-graduate schools of Hygiene and Preventive Medicine (Sicily & Calabria)	Medical residents	Anonymous survey, no s.	SIV uptake previous five years. Reasons for adherence or refusal.	73, 94.5%	Coverage 20.3% previous year, 27.5% current year. Compliance motivated with desire to not spread infection to others; refusal mainly due to low perception of risk from disease.
[19]	Apr 2012 – Jun 2012	Eighteen Italian post-graduate schools	Medical residents	Anonymous web-based survey, no s.	SIV uptake last 3 years. Reasons for adherence or refusal.	10396, 24.1%	Coverage declining with time from 21.7% to 11.9%. VA mostly for protection of themselves and others; refusal mainly motivated with low risk perception
[20]	Dec 2013	Teaching hospital (Rome, Lazio)	All personnel	Anonymous survey, cluster (units) s.	Compliance to annual SIV. Reasons for adherence or refusal.	191, 90.8%	6.8% compliance to annual SIV. Patient protection main reason for VA; low risk perception main driver for refusal; missed revaccination motivated by low SIV efficacy (after 2009 pandemic). Higher OR for physicians, greater seniority
[21]	February-March 2016	GPs[2] of Taranto	GPs[2]	Cross-sectional study	SIV among GPs[2] and factors influencing their adherence to the vaccinations.<br>Tool: self-administered web-based standardized questionnaire.	229/471 (48.6%)<br><br>[229 (M 21%/F 79%)]	SIV 2015/2016: 76.4% (175/229). >=900 patients increased the SIV likelihood. 79.9% of GPs[2] prefer to use the adjuvated vaccines on patients aged >64. Motivational factors pro-vaccination: 53.1 % (93/175) their own health protection; 45.1% (79/175) to protect the health of their own patients; 25.4% (62/175) to protect their family and friends. GPs[2] without SIV: 58% (30/51) influenza as non-dangerous pathology; 5.9% (3/51) vaccine could be risky; 35.3% to avoid reactions.
[22]	Oct 2013 – Feb 2014	Teaching hospital and LHU[1] (Genoa, Liguria)	All personnel	Anonymous survey, no s.	SIV uptake last 6 years. Reasons for adherence or refusal.	8248, 10.1%	Annual coverage 25.6-31.4%. 12.5% compliance through whole period. Protection of themselves and family main

							reason for VA. Higher OR for physicians, belief in importance in annual SIV, trust in SIV safety; lower OR for mistrust in pharma companies
[23]	2005-6 flu season	Paediatric hospital (Florence, Tuscany)	Nurses	Intervention study and anonymous survey, no s.	SIV uptake after active no-cost offer. Reasons for adherence or refusal.	327, unknown	30.3% coverage. Patient protection (62%) main driver, followed by self and family protection, and gratuity. Perceived futility of SIV main reason for refusal.
[24]	Nov 2009 – Mar 2011	Fifty-one hospitals (Apulia, Italy)	All personnel	Anonymous survey, no s.	SIV uptake	2198 respondents (total unknown)	24.8% coverage. Highest OR for females, physicians, being offered SIV from GP[2] or occupational health physician
[25]	Apr 2014	University of Bari, Italy	Students attending medical and paramedical degree courses	Case-control study	Determinants of vaccination compliance.<br>Tool: online anonymous questionnaire administered.	NR [669 (M 46% / F 54%)]	SIV associated with: 1. being invited from the University; 2. opinion that vaccine is safe and useful; 3. specific training about influenza vaccination during the course; 4. considering themselves as at a major risk of flu-related complication.
[26]	2017-2018 and 2010-2013 flu seasons	A large Teaching Hospital (Molinette) selected from *Azienda Ospedaliera Universitaria "Città della salute e della Scienza,"* a complex of four interconnected hospitals.	HP	Datasets from hospital registers, national and regional reports, subsequently merged and analysed.	Excess absenteeism during 2017-2018 severe influenza season, compared with 2010-2013 moderate flu seasons	5287 (approximately 45% of the all employees) [M 26.3% / F 73.7%]	Increased absenteeism among HP during the epidemic period of severe season (weeks 42-17) in comparison with non-epidemic periods (weeks 18-41), the absolute increase correlated with a relative increase of 70% (from 4.05 to 6.68 days/person). Less excess of absenteeism in vaccinated HP vs. non-vaccinated (1.74 vs 2.71 days/person).
[27]	October 2012 - April 2014	HP in Europe (14 EU countries), Data from Italy (n=251).	HP	14 European countries, including Greece, Italy and Romania	To explore HP attitudes and behaviours towards vaccination for a number of VPDs. Tool: online anonymous questionnaire.	5424/5553 (97.7%)	HP considered influenza (86.4%) as highest risk disease for occupational exposure in the workplace. 43.8% without

						[5424 (M 19.1 / F 80.9%)]	SIV in the last 10 years, 65.6% not vaccinated against pandemic influenza in 2009. HP from Italy and Slovenia had the highest probabilities of not believing in vaccinations in relation to those from Sweden. The main barriers for SIV: belief of challenging natural immunity by contracting the disease (19.5%), side effect concerns (14.5%).
[28]	May - July 2019	Infection control team (ICT). A collaborative survey by European Committee of infection Control (EUCIC) members.	Italy one out of 56 respondent countries. ICT members (physicians, nurses) as main SIV motivators among HP.	Cross-sectional study	To examine the opinion of ICT member about SIV and related factors affecting their perceptions.<br>Tool: self-administered online survey.	NR [899 (M 32%/F 68%)]	SIV 100% among ICT in Finland, Portugal, Norway, Israel, vs lowest rates in Italy (68%) and Turkey (39%). Most significant factors for ICT members' SIV: 1. personal influenza vaccine experience (49%); 2. scientific authorities (48%); 3. being members of ICT.
[29]	Sep 2018 – Oct 2018	Twenty-eight nursing homes (Tuscany)	Nurses, physiotherapists, aides, educators, nonclinical staff	Anonymous survey, no s.	SIV uptake previous 2 years and intention current year. Standardized measures of health literacy and vaccine confidence	710 respondents (total unknown)	16% past uptake, 28.4% planned. Significant association w/ vaccine confidence, living w/ elderly people, chronic diseases; no association w/ health literacy.
[30]	A. Nov 2019 – Dec 2019<br>B. Summer 2020	A. Eight nursing homes<br>B. 111 nursing homes, including the 8 from A (Tuscany)	Not specified	Anonymous survey, no s.<br>A. Intervention study (nudge)<br>B. Cross-sectional study (intervention vs control)	A. SIV intention. Qualitative analysis of reasons.<br>B. SIV uptake previous 2 years and intention current year. Standardized measure of vaccine confidence	A. 527, 40.2%<br>B. 2135, 47.8%	A. 51.8% planning to receive SIV. Risk perception for themselves and contacts as main reason for VA (83%) and refusal (55%).<br>B. Similar coverage before intervention (22-23%); higher uptake (28% vs 20%), intention (38% vs 31%) and vaccine confidence in intervention group
[31]	Nov 2019 – Dec 2019	Teaching hospital (Milan, Lombardy)	All personnel, including administrative staff	Analysis of administrative data + anonymous survey of refusals	SIV uptake.	3405, NA	21.5% coverage, increased from previous year after introduction of on-site offer. Refusal associated w/ nurses, female sex, young age, low perception of disease severity, concern for vaccine risks, disbelief in vaccine efficacy

[32]	Apr 2019 – Jun 2019	Teaching hospital (Rome, Lazio)	Nurses and midwives	Anonymous web-based survey, convenience sample	SIV uptake last 3 years and intention next year. Opinions and reasons about SIV	66, 92.4%	31-57% coverage last 3 years, 84% intention. Higher VA and better opinions about SIV in HP involved in seasonal vaccination campaign.
[33]	Sep 2018 – Nov 2018	Eight random-selected hospitals in Campania and Calabria	All personnel from critical care units	Anonymous survey, random s.	SIV uptake. Perceived risks and benefits	967, 54.9%	35.8% coverage. Higher rate for physicians. Perceived low risk of infection main reason for refusal.
[34]	2019-2020, 2020-2021 and 2021-2022 flu seasons	IRCCS Ospedale Policlinico San Martino, Genoa	HP	Retrospective, single-center study	SIV coverage rates in the 2019-2020, 2020-2021 and 2021-2022 seasons	6194 (M 2120 / F 4074)	SIV coverage was below the recommended target in all seasons, with a sharp increase was observed in 2020/2021 (40.9%), from 12.8% in 2019/2020 and 23% in 2021/2022. In 2019/2020 and 2021/2022 seasons, physicians were higher vaccinated.
[35]	Jun 2018 – Aug 2018	Pediatric university hospital, selected units (Florence, Tuscany)	Physicians, nurses, assistants, technicians	Anonymous survey, no s.	SIV uptake last year. Reasons for adherence of refusal	31% resp. rate (108 respondents)	~20% coverage. Patient protection as main reason for VA; refusal mostly motivated with perceived low risk of disease
[36]	Nov 2020 – Dec 2020	Teaching hospital (Milan, Lombardy)	All personnel, incl. administrative staff	Analysis of administrative data + anonymous survey of recipients, no s.	SIV uptake. Reasons for adherence	Unreported	43.1% coverage (21.5% 2019). Importance of vaccines for prevention and patient protection as main reasons for VA.
[37]	Oct 2016 – Dec 2016	South Tyrolean Health Service	All personnel, incl. administrative staff	Anonymous web-based survey, no s.	SIV uptake last 4 years. Reasons for adherence of refusal	9633, 42.4%	10.4% coverage. Higher OR for males, physicians, unhealthy lifestyle factors. Perceived low risk of disease as main driver of refusal.
[38]	2015	Autonomous province of Trento	Occupational physicians	Anonymous survey, convenience sample	SIV uptake previous year. Perception of infection risks, severity and vaccine risks, reasons for refusal	105, 87.6%	46.7% coverage. Lack of time and belief in immunity from previous vaccinations as main reasons for refusal. Higher OR for belief in vaccine efficacy.

[39]	May 2019	Palermo University	Nursing students	Cross-sectional study	To ascern the determinants of vaccination uptake. Tool: anonymous paper questionnaire.	403/409 (98.5%) (M 35%/F 65%)	SIV 2019: 21%; intention to vaccinate in next season: 46.6%. Students with a perceived medium-high state of health more likely to vaccinate in the next vaccination campaign. Low risk perception of infection (35.5%); infection not a risk for family/friends (9%); infection not a risk for patients (3%). Forgetting to be vaccinated (16.4%). Concerns about efficacy (10%). Lack of recommendation by the facility (26%).
[40]	March - June 2018	University hospital "G. Martino" of Messina	HP	Cross-sectional study	Self-completion questionnaire based on Attachment 3 of Ministerial Circular 25233 of 18 August 2017	822 health care workers (324 males and 498 females with an age of 49.5 ± 10.5 SD)	Higher vaccination coverages were found for females, physicians and the clinical area and - for influenza vaccination - in the older age groups.
[41]	2017-8 flu season	Four teaching hospitals (Rome, Lazio)	All personnel	Multicentric intervention study; analysis of administrative data	SIV uptake.	12226, NA	4.23% - 12.97% coverage. Higher rates in hospitals conducting multiple actions in education, promotion and access.
[42]	2015	Pediatric hospital (Florence, Tuscany)	Nurses (inc. students), pediatricians, technicians, radiologists, pharmacists	Anonymous survey, recruiting strategy not reported	SIV uptake previous 5 years.	860, 20%	68.3% never vaccinated in the last 5yy. Lower rates among nurses.
[43]	October 2016-January 2018	Bari Policlinic Hospital, Italy	HP	Cross-sectional study	OSV[3] strategy outcome in 2017/2018 compared to SIV among HP in the ad hoc clinic-based previous season.	NR<br>295 (M 49.5% F 50.5)<br>482 (M 49.2%/ F 50.8%)	Vaccination coverage in the 2016/17 influenza season: 8.7%.<br>Vaccination coverage in the 2017/18 season: 14.2%.<br>OSV[3] led to an increase of vaccinated HP compared to the classical vaccination clinic approach.

[1] LHU(s): Local Health Unit(s); [2] GP(s): General Practitioner(s); [3] OSV: On-Site Vaccination.

Study on influenza vaccination uptake among health professionals in Italy, 1990-2022.

1. Albano, L.; Matuozzo, A.; Marinelli, P.; Di Giuseppe, G. Knowledge, attitudes and behaviour of hospital health-care workers regarding influenza A/H1N1: a cross sectional survey, *BMC Infect Dis*, **2014**, 14, 208. doi: 10.1186/1471-2334-14-208.
2. Alicino, C.; et al. Influenza vaccination among healthcare workers in Italy. *Hum Vaccin Immunother*, **2015**, 11(1), 95–100. doi: 10.4161/hv.34362.
3. Amodio, E.; et al. Are medical residents a core group for future improvement of influenza vaccination coverage in health-care workers? A study among medical residents at the University Hospital of Palermo (Sicily). *Vaccine*, **2011**, 29 (45), 8113–8117. doi: 10.1016/j.vaccine.2011.08.033.
4. Amodio, E.; Anastasi, G.; Marsala, M.G.L.; Torregrossa, M.V.; Romano, N.; Firenze, A. Vaccination against the 2009 pandemic influenza A (H1N1) among healthcare workers in the major teaching hospital of Sicily (Italy). *Vaccine*, **2011**, 29(7), 1408–1412. doi: 10.1016/j.vaccine.2010.12.041.
5. Amodio, E.; et al. Influenza vaccination among healthcare workers and absenteeism from work due to influenza-like illness in a teaching hospital in Palermo. *Italian Journal of Public Health*, **2010**, 7(3).
6. Arghittu, A.; et al. Flu Vaccination Attitudes, Behaviours, and Knowledge among Health Workers. *Int J Environ Res Public Health*, **2020**, 17(9), 3185. doi: 10.3390/ijerph17093185.
7. Ballada, D.; et al. Attitudes and behavior of health care personnel regarding influenza vaccination. *Eur J Epidemiol*, **1994**, 10(1), 63–68. doi: 10.1007/BF01717454.
8. Barbara, A.; La Milia, D.I.; Di Pumpo, M.; Tognetto, A.; Tamburrano, A.; Vallone, D.; Viora, C.; Cavalieri, S.; Cambieri, A.; Moscato, U.; Berloco, F.; Damiani, G.; Ricciardi, W.; Capelli, G.; Laurenti, P. Strategies to Increase Flu Vaccination Coverage among Healthcare Workers: A 4 Years Study in a Large Italian Teaching Hospital. *Vaccines*. **2020**, 13, 8(1), 85. doi: 10.3390/vaccines8010085.
9. Barbadoro, P.; et al. Gender, Socioeconomic, and Health Characteristics Associated with Influenza Vaccination Coverage (VC) among Italian Healthcare Workers: Secondary Analysis of a National Cross-Sectional Survey. *Healthcare*, **2020**, 8(3), Art. n. 3. doi: 10.3390/healthcare8030298.
10. Bianchi, F.P.; Tafuri, S.; Spinelli, G.; Carlucci, M.; Migliore, G.; Calabrese, G.; Daleno, A.; Melpignano, L.; Vimercati, L.; Stefanizzi, P. Two years of on-site influenza vaccination strategy in an Italian university hospital: main results and lessons learned. *Human Vaccines & Immunotherapeutics* **2022,** 18, 1, 1993039.
11. Bonaccorsi, G.; et al. Predictive factors associated with the acceptance of pandemic and seasonal influenza vaccination in health care workers and students in Tuscany, Central Italy. *Hum. Vaccines Immunother.*, **2013** 9(12), 2603–2612. doi: 10.4161/hv.26036.
12. Bonaccorsi, G.; et al. Beliefs and Opinions of Health Care Workers and Students Regarding Influenza and Influenza Vaccination in Tuscany, Central Italy. *Vaccines*, **2015**, 3(1), Art. n. 1. doi: 10.3390/vaccines3010137.
13. Brunelli, L.; Antinolfi, F.; Malacarne, F.; Cocconi, R.; Brusaferro, S. A Wide Range of Strategies to Cope with Healthcare Workers' Vaccine Hesitancy in A North-Eastern Italian Region: Are They Enough? *Healthcare*, **2021**, 9(1). doi: 10.3390/healthcare9010004.
14. Campagna, M.; et al. Current preventive policies and practices against Vaccine-Preventable Diseases and tuberculosis targeted for workers from hospitals of the Sardinia Region, Italy. *J Prev Med Hyg*, **2016,** 57(2), E69–E74.
15. Castella, A.; Argentero, P.A.; Lanszweert, A. Factors influencing uptake of influenza vaccination in healthcare workers. Findings from a study in a general hospital. *Ann Ig*, **2009**, 21(1), 35–40.
16. Chittano Congedo, E.; Paladino, M.E.; Riva, M.A; Belingheri, M. Adherence, Perception of, and Attitude toward Influenza and Flu Vaccination: A Cross-Sectional Study among a Population of Future Healthcare Workers. *Int J Environ Res Public Health*. **2021**, 18(24), 13086. doi: 10.3390/ijerph182413086.
17. Costantino, C.; et al. Influenza vaccination coverage among medical residents. An Italian multicenter survey. *Hum. Vaccines Immunother* **2014,** 10(5), 1204–1210. doi: 10.4161/hv.28081.
18. Costantino, C.; et al. Attitudes, behaviours and perceptions of Italian General Practitioner trainees towards influenza vaccination in Western Sicily (Italy). *Italian Journal of Public Health*, **2012,** 9(1).
19. Costantino, C.; et al. Knowledge, attitudes and behaviors regarding influenza vaccination among Hygiene and Preventive Medicine residents in Calabria and Sicily. *EuroMediterranean Biomedical Journal*, **2012,** 7(17), 77–83.
20. Cozza, V.; Alfonsi, V.; Rota, M.C.; Paolini, V.; Ciofi degli Atti, M.L. Promotion of influenza vaccination among health care workers: findings from a tertiary care children's hospital in Italy. *BMC Public Health*, **2015,** 15(1), Art. n. 1. doi: 10.1186/s12889-015-2067-9.
21. Desiante, F.; Caputi, G.; Cipriani, R.; et al. Assessment of coverage and analysis of the determinants of adherence to influenza vaccination in the general practitioners of Taranto. *Ann Ig*. **2017**;29(4):256-263. doi:10.7416/ai.2017.2157
22. Durando, P.; et al. Determinants of adherence to seasonal influenza vaccination among healthcare workers from an Italian region: Results from a cross-sectional study. *BMJ Open*, **2016,** 6(5). doi: 10.1136/bmjopen-2015-010779.
23. Festini, F.; Biermann, K.P.; Neri, S.; Reali, M.F.; de Martino, M. Influenza vaccination of nurses in an Italian pediatric hospital: effects on absenteeism and on costs, factors associated with vaccine uptake and analysis of personal motivations. A prospective cohort study. *Assistenza Infermieristica E Ricerca*, **2007**, 26(1), 5–13.
24. Fortunato, F.; Tafuri, S.; Cozza, V.; Martinelli, D.; Prato, R. Low vaccination coverage among Italian healthcare workers in 2013. *Human Vaccines & Immunotherapeutics*, **2015** 11(1), 133–139. doi: 10.4161/hv.34415.
25. Gallone, M.S.; Gallone, M.F.; Cappelli, M.G.; et al. Medical students' attitude toward influenza vaccination: Results of a survey in the University of Bari (Italy). *Hum Vaccin Immunother*. **2017,** 13(8), 1937-1941. doi:10.1080/21645515.2017.1320462.

26. Gianino, M.M.; Kakaa, O.; Politano, G.; Scarmozzino, A.; Benso, A.; Zotti, C.M. Severe and moderate seasonal influenza epidemics among Italian healthcare workers: A comparison of the excess of absenteeism. *Influenza Other Respir Viruses*. **2021,** 15(1), 81-90. doi: 10.1111/irv.12777.
27. Karnaki, P.; Baka, A.; Petralias, A.; et al. Immunization related behaviour among healthcare workers in Europe: Results of the HProImmune survey. *Cent Eur J Public Health*. **2019,** 27(3), 204-211. doi:10.21101/cejph.a5514.
28. Keske, Ş.; Mutters, N.T.; Tsioutis, C.; Ergönül, Ö.; EUCIC influenza vaccination survey team. Influenza vaccination among infection control teams: A EUCIC survey prior to COVID-19 pandemic. *Vaccine*. **2020,** 38(52), 8357-8361. doi:10.1016/j.vaccine.2020.11.003.
29. Lorini C.; et al. Health literacy, vaccine confidence and influenza vaccination uptake among nursing home staff: a cross-sectional study conducted in Tuscany. *Vaccines*, **2020**, 8(2), 154.
30. Lorini C.; et al. Promoting Influenza Vaccination among Staff of Nursing Homes According to Behavioral Insights: Analyzing the Choice Architecture during a Nudge-Based Intervention. *Vaccines*, **2020,** 8(4). doi: 10.3390/vaccines8040600.
31. Maffeo M.; et al. 2019 Influenza Vaccination Campaign in an Italian Research and Teaching Hospital: Analysis of the Reasons for Its Failure. *Int J Environ Res Public Health*, **2020**, 17(11), 3881. doi: 10.3390/ijerph17113881.
32. Mellucci, C.; et al. Vaccine Hesitancy among Master's Degree Students in Nursing and Midwifery: Attitude and Knowledge about Influenza Vaccination. *International Journal of Environmental Research and Public Health*, **2020**, (17). doi: 10.3390/ijerph17197191.
33. Napolitano, F.; Bianco, A.; D'Alessandro, A.; Papadopoli, R.; Angelillo, I.F. Healthcare workers' knowledge, beliefs, and coverage regarding vaccinations in critical care units in Italy. *Vaccine*, **2019,** 37(46), 6900–6906. doi: 10.1016/j.vaccine.2019.09.053.
34. Ogliastro, M.; Borghesi, R.; Costa, E.; Fiorano, A.; Massaro, E.; Sticchi, L.; Domnich, A.; Tisa, V.; Durando, P.; Icardi, G.; Orsi, A. Monitoring influenza vaccination coverage among healthcare workers during the COVID-19 pandemic: a three-year survey in a large university hospital in North-Western Italy. *J Prev Med Hyg*. **2022**, 27, 63(3), E405-E414. doi: 10.15167/2421-4248/jpmh2022.63.3.2700.
35. Paoli, S.; et al. Assessing Vaccine Hesitancy among Healthcare Workers: A Cross-Sectional Study at an Italian Paediatric Hospital and the Development of a Healthcare Worker's Vaccination Compliance Index. *Vaccines*, **2019,** 7(4). doi: 10.3390/vaccines7040201.
36. Perrone, P.M.; et al. Influenza Vaccination Campaign during the COVID-19 Pandemic: The Experience of a Research and Teaching Hospital in Milan. *Int J Environ Res Public Health*, **2021,** 18(11), 5874. doi: 10.3390/ijerph18115874.
37. Rabensteiner, A.; Buja, A.; Regele, D.; Fischer, M.; Baldo, V. Healthcare worker's attitude to seasonal influenza vaccination in the South Tyrolean province of Italy: barriers and facilitators. *Vaccine*, **2018,** 36(4), 535–544. doi: 10.1016/j.vaccine.2017.12.007.
38. Riccò, M.; Cattani, S.; Casagranda, F.; Gualerzi, G.; Signorelli C. Knowledge, attitudes, beliefs and practices of Occupational Physicians towards seasonal influenza vaccination: a cross-sectional study from North-Eastern Italy. *J Prev Med Hyg*, **2017,** 58(2), E141–E154.
39. Santangelo, O.E.; Provenzano, S.; Firenze, A. Factors influencing flu vaccination in nursing students at Palermo University. *J Prev Med Hyg*. **2021,** 61(4), E563-E567. doi:10.15167/2421-4248/jpmh2020.61.4.1426.
40. Squeri, R.; La Fauci, V.; Picerno, I.A.M.; Trimarchi, G.; Cannavò, G.; Egitto, G.; Cosenza, B.; Merlina, V.; Genovese, C. Evaluation of Vaccination Coverages in the Health Care Workers of a University Hospital in Southern Italy. *Ann Ig*. **2019**, 31(2Supple 1), 13-24. doi: 10.7416/ai.2019.2273.
41. Tognetto, A.; et al. Seasonal influenza vaccination among health-care workers: the impact of different tailored programs in four University hospitals in Rome. *Hum. Vaccines Immunother.*, **2020,** 16(1), 81–85. doi: 10.1080/21645515.2019.1632684.
42. Tomboloni, C.; et al. Knowledge, attitude and disinformation regarding vaccination and immunization practices among healthcare workers of a third-level paediatric hospital. *Ital J Pediatr*, **2019,** 45, 104. doi: 10.1186/s13052-019-0684-0.
43. Vimercati, L.; Bianchi, F.P.; Mansi, F.; et al. Influenza vaccination in health-care workers: an evaluation of an on-site vaccination strategy to increase vaccination uptake in health professionals of a South Italy Hospital. *Hum Vaccin Immunother*. **2019,** 15, 12, 2927-2932. doi:10.1080/21645515.2019.1625645.
